# sTREM2 Differentially Affects Cytokine Expression in Myeloid-Derived Cell Models via MAPK–JNK Signaling Pathway

**DOI:** 10.3390/biology13020087

**Published:** 2024-01-30

**Authors:** Ryan Arsenault, Steven Marshall, Patrick Salois, Qiao Li, Wandong Zhang

**Affiliations:** 1 Human Health Therapeutics Research Centre, National Research Council of Canada, Ottawa, ON K1A 0R6, Canada or rarse078@uottawa.ca (R.A.); patrick.salois@nrc-cnrc.gc.ca (P.S.); 2 Department of Cellular and Molecular Medicine, Faculty of Medicine, University of Ottawa, Ottawa, ON K1H 8M5, Canada; smars042@uottawa.ca

**Keywords:** TREM2, soluble TREM2 (sTREM2), THP-1 cells, monocytes, macrophage, innate immune response, inflammatory response, cytokine expression, MAPK–JNK signaling, NLRP3 inflammasome

## Abstract

**Simple Summary:**

TREM2 is a critical innate immune receptor belonging to the immunoglobulin superfamily and plays an important role in regulating the function of innate immune cells, such as microglia and macrophages. TREM2 mutations or dysregulation of TREM2 signaling are associated with the risk of neurodegenerative diseases, such as Alzheimer’s disease. sTREM2 is a soluble form of TREM2 shed from the cell surface TREM2 receptor and has a short half-life (~4 h), but its roles still remain largely uncharacterized. Our study has found that sTREM2 can induce the expression of inflammatory cytokines in THP-1 monocytes during the time course from 2 to 8 h but up-regulates the expression of anti-inflammatory cytokines at later time points. sTREM2 has differential effects on cytokine expression in macrophages with more stimulating effects on M0 macrophages, less of an effect on M2 macrophages, and some inhibitory effects on M1 macrophages at early time-points. Characterization of several signaling pathways found that sTREM2-mediated cytokine expression mainly occurs via MAPK–JNK signaling in cells. Our study suggests that sTREM2 has differential effects on cytokine expression in THP-1 monocytes and macrophages and that MAPK–JNK signaling is mainly involved in sTREM2-mediated cytokine expression in cells.

**Abstract:**

TREM2 is a critical innate immune receptor primarily expressed on myeloid-derived cells, such as microglia and macrophages. Mutations in TREM2 are linked to several neurodegenerative diseases including Alzheimer’s disease (AD). TREM2 can be cleaved from the cell membrane and released as soluble TREM2 (sTREM2). sTREM2 levels are shown to peak prior to AD, with its levels fluctuating throughout disease progression. However, the mechanism by which sTREM2 may affect innate immune responses is largely uncharacterized. In this study, we investigated whether sTREM2 can induce inflammatory response in myeloid-derived THP-1 monocytes and macrophages and characterized the signaling mechanisms involved. Our results show that sTREM2 was capable of stimulating the expression of several inflammatory cytokines in THP-1 cells throughout the time course of 2 h to 8 h but inducing anti-inflammatory cytokine expression at later time points. A TREM2 antibody was capable of inhibiting the expression of some cytokines induced by sTREM2 but enhancing others. The complex of sTREM2/TREM2 antibody was shown to enhance IL-1β expression, which was partially blocked by an NLRP3 specific inhibitor, indicating that the complex activated the NRLP3 inflammasome pathway. sTREM2 was also shown to have differential effects on cytokine expression in M0, M1, and M2 macrophages differentiated from THP-1 cells. sTREM2 has a more stimulating effect on cytokine expression in M0 macrophages, less of an effect on M2 macrophages, and some inhibitory effects on cytokine expression in M1 macrophages at early time points. Analyses of several signaling pathways revealed that sTREM2-induced expression of cytokines occurs mainly through MAPK–JNK signaling. Our work reveals differential effects of sTREM2 on cytokine expression profiles of THP-1 cells and macrophages and demonstrates that the MAPK–JNK signaling pathway is mainly responsible for sTREM2-induced cytokine expression.

## 1. Introduction

TREM2 (Triggering Receptor Expressed on Myeloid Cells 2) is an innate immune receptor predominantly expressed on myeloid lineage cells such as macrophages and microglia. Belonging to the immunoglobulin (Ig) superfamily, TREM2 is a 230 amino acid polypeptide consisting of a single-pass transmembrane receptor that also contains a single extracellular V-type Ig domain followed by a short stalk leading to a single transmembrane helix [1] where it interacts with DNAX-activation protein 12 (DAP12), encoded by *TYROBP* (*tyrosine kinase-binding protein*), to mediate downstream signaling pathways [2]. TREM2 is associated with DAP12, which contains an immunoreceptor tyrosine-based activation motif (ITAM). Upon ligand binding by TREM2, tyrosine residues within ITAM become phosphorylated, recruiting and phosphorylating spleen tyrosine kinase (Syk) to activate downstream signaling molecules such as extracellular signal-regulated protein kinase (ERK), phosphatidylinositol 3-kinase (PI3K), and phospholipase Cγ (PLCγ) [3]. TREM2 terminates with a short cytoplasmic tail lacking any known trafficking motifs [4]. Although expressed primarily on the membrane of myeloid-derived cells, TREM2 can shed its extracellular domain from the cell membrane as a soluble form (sTREM2), but the complete functional outcome of sTREM2 remains largely unknown. Shedding of membrane-bound TREM2 is mediated by α-secretases A Disintegrin and Metalloproteinase 10/17 (ADAM10/17) to cleave TREM2 at the H157–S158 bond releasing the N-terminal fragment of TREM2, sTREM2, into the extracellular space [5]. The physiological functions of TREM2 are relatively well characterized; however, the complete roles of its soluble form both physiologically and pathologically still remain elusive. Interestingly, it was shown that both TREM2 and sTREM2 bind oligomeric Aβ and that TREM2 deficiency impairs Aβ degradation in primary microglial cultures and mouse brains [6], suggesting sTREM2 may act as a scavenger receptor capable of recruiting cells and acting as a sort of chemokine.

Under normal physiological conditions, TREM2 signaling in microglia promotes cell survival, proliferation, activation, phagocytosis, and inflammation regulation [7]. TREM2 can bind a diverse array of ligands including bacterial lipopolysaccharides (LPS), Aβ peptides, and damage-associated molecular patterns (DAMPs), such as phospholipids [8], lipids exposed during axonal injury [9], and ApoE2/E3/E4 [10]. TREM2 in mice is shown to be involved in synaptic pruning [11], and mice deficient in TREM2 showed reduced numbers of synapse at 4 weeks of age [12]. Atagi et al. [10] uncovered a functional relationship between TREM2 and ApoE, in which ApoE increases microglial phagocytosis in a TREM2-dependent manner. It was also shown that each isoform binds TREM2 with high affinity and induces differential levels of phosphorylated Syk, with ApoE2 and ApoE3 binding more readily to TREM2 than ApoE4 [10]. Contrary to this, ApoE4 stimulates Syk activation to a greater extent than either ApoE2 or ApoE3 [13].

The soluble form of TREM2, sTREM2, is believed to play critically important roles in the development and progression of AD. Some studies have shown that sTREM2 is neuroprotective by stimulating microglial recruitment, activation, and phagocytosis of Aβ plaques and by blocking, reversing, or preventing neurotoxicity [14]. sTREM2 has also been shown to reduce Aβ pathology and to improve synaptic functions by modulating microglial activity in the 5xFAD mouse model [15]. Not only has the research shown that sTREM2 directly interacts with AD pathologies, but its levels in cerebrospinal fluid (CSF) were reportedly elevated in AD patients compared to controls, and it showed a correlation to total tau and phosphorylated tau but not to Aβ [16,17]. Moreover, sTREM2 was seen to increase in the preclinical stages of AD [17,18] and then decrease at early symptomatic stages [17], supporting the idea that sTREM2 plays a critical role in AD disease progression. sTREM2 may also act as a second messenger to allow crosstalk between microglia to coordinate an appropriate response. Interestingly, CSF levels of sTREM2 differed between TREM2 variants, whereas the R47H variant had higher CSF sTREM2 and L211P variant had lower CSF sTREM2 [17], suggesting that mutations in TREM2 influence TREM2 release/sTREM2 levels or that shedding may be mediated by intrinsic factors. Moreover, sTREM2 signaling in microglia was shown to increase survival [18], and sTREM2 may be capable of initiating cytokine expression in cells. Our study reveals that sTREM2 has differential effects on cytokine profiles of THP-1 cells and macrophages and that sTREM2-induced cytokine expression in cells is mainly mediated via the MAPK–JNK signaling pathway.

## 2. Materials and Methods

### 2.1. Cell Cultures

THP-1 line (human monocytic leukemia cell line) was obtained from ATCC (TIB-202^™^). Cells were cultured in suspension in RPMI 1640 (Wisent Bioproducts, Saint-Jean-Baptiste, QC, Canada) supplemented with 10% fetal bovine serum (FBS), 1X penicillin/streptomycin (ThermoFisher Scientific, Waltham, MA, USA), and 1X L-glutamine (ThermoFisher Scientific). Cells were maintained in an incubator at 37 °C and 5% CO_2_. Media was replaced every 2–3 days by fresh complete RPMI 1640 media, and cells were passaged once cell concentrations exceeded 1 × 10^6^/mL. Cells were discarded after passage 25. Passages 5–15 were used throughout this study.

### 2.2. Treatment of THP-1 Cells with sTREM2

THP-1 monocytes were counted on a hemocytometer. The cells (3.6 million cells) were centrifuged at 300× *g* for 7 min at room temperature. Cell culture media was then removed, and cells were resuspended in RMPI1640 containing 1% FBS. THP-1 cells were then pipetted onto a 24-well plate at 150,000 cells per well. Human soluble TREM2 (sTREM2 protein) (Sino Biological, Beijing, China, #11084-H08H; His-tagged, expressed, and purified from HEK293 cells) was purchased and reconstituted according to manufacturer’s instructions in sterile water and stored at −20 °C. Plated cells were then treated with 0.1 μg/mL or 1.0 μg/mL of sTREM2. Negative control cells were treated with sterile water, and 0.1 μg/mL lipopolysaccharide (LPS) (Sigma, St. Louis, MI, USA) was used as a positive control to induce inflammatory cytokine expression. Cells were harvested at 2, 4, 6, and 8 h post treatment and supernatants were collected and stored at −80 °C for later RT-qPCR or/and cytokine ELISA.

Rat monoclonal IgG_2B_ antibody against human TREM2 was purchased from R&D Systems (#MAB17291; Protein A or G purified, endotoxin-free). The antibody was reconstituted according to manufacturer’s instructions and was stored at −20 °C. THP-1 cells were plated as described previously, and cells were treated with either 0.1 μg/mL antibody, 0.1 μg/mL sTREM2, or both 0.1 μg/mL sTREM2 and antibody. Antibody and sTREM2 were pre-incubated together at 37 °C in 1% FBS RPMI1640 media for 30 min prior to adding to THP-1 cells. Cells were harvested at 2, 4, 6 and 8 h post treatment, and supernatants were collected and stored at −80 °C for later ELISA. The cells were also treated with both 0.1 μg/mL sTREM2 + antibody + MCC950 (7.5 nM) (a specific NLRP3 inhibitor, Selleckchem.com (https://www.selleckchem.com/ (Accessed on 9 May 2022)).

Chemical inhibitors were purchased from Selleck Chemical (Selleckchem.com; https://www.selleckchem.com/ (Accessed on 9 May 2022)) and reconstituted according to the manufacturer’s instructions in order to target major signaling pathways. Prior to treatment, the IC50 of each inhibitor was tested on cultured cells for 8 h to ensure it did not affect cell viability using the dye exclusion test. SP600125 (#S1460), a broad spectrum JNK inhibitor for JNK1, JNK2, and JNK3 with an IC50 of 40 nM and 90 nM, respectively, was used to target the JNK–MAPK pathway. Piceatannol (S3026), a natural stilbene and selective Syk inhibitor with an IC50 of 15 μM, was used to target TREM2–Syk signaling. MG-132 (#S2619), a potent, wel—known proteasome inhibitor with an IC50 of 0.1 μM, was used to target NFκB activation. Cells were plated as previously described in 1% FBS RPMI1640, treated with either 0.1 μg/mL sTREM2; IC50 of either SP00125, MG-132, or Piceatannol; or both 0.1 μg/mL sTREM2 and IC50 of either inhibitor. Prior to the addition of sTREM2, cells were pre-incubated with the inhibitor for 10 min. Control cells were treated with DMSO. Cells were harvested at 6 h post treatment and supernatants were collected and stored at −80 °C for later ELISA assays. MCC950 (Selleck chemicals, #S7809) was used to inhibit NLRP3 inflammasome activation. MCC950 at 7.5 nM was incubated with THP-1 cells for 10 min prior to the addition of 0.1 μg/mL sTREM2, 0.1 μg/mL TREM2 antibody, or both. At 6 h, cells were harvested, and the expression of cytokines was assessed using RT-qPCR.

### 2.3. Differentiation and Polarization of THP-1 Monocytes into Macrophages

The desired number of THP-1 monocytes was centrifuged at 300× *g* for 7 min at room temperature and resuspended in monocyte attachment media (PromoCell, Stemcell technologies) (https://www.stemcell.com/ (Accessed on 9 September 2021)) to a final concentration of 1.5 × 10^6^ cells/mL. Cells (100 μL containing 150,000) were pipetted per well into a 96- or 24-well plate and allowed to sit at room temperature for 10 min The plate was then transferred to an incubator and allowed to incubate for 1.5 h at 37 °C and 5% CO_2_. Following this brief incubation, the attachment media was replaced with fresh RPMI1640 substituted with 10% heat-inactivated FBS (FBS was heat-inactivated at 56 °C with moderate mixing for 30 min) and supplemented with 25 ng/mL PMA (Phorbol 12-myristate 13-acetate) (Sigma Aldrich; Oakville, ON, Canada) (https://www.sigmaaldrich.com/CA/en (Accessed on 1 October 2021)). The plate was transferred to an incubator at 37 °C and 5% CO_2_ for 72 h for THP-1 cells to differentiate. Differentiated cells were then washed twice with RPMI1640 10% FBS and allowed to rest in media for 24 h at 37 °C and 5% CO_2_. Following a brief resting period, cells were then polarized for 48 h at 37 °C and 5% CO_2_ to M0, M1, and M2 macrophages. M0 macrophages using RPMI1640 as described above were supplemented with no additional cytokines. M1 macrophages were polarized using RMPI1640 supplemented with 10 pg/mL LPS (Sigma Aldrich; Oakville, ON, Canada) (https://www.sigmaaldrich.com/CA/en (Accessed on 1 October 2021)) and 25 ng/mL IFN-γ (StemCell Technologies, #78141; Vancouver, BC, Canada; https://www.stemcell.com/ (Accessed on 9 September 2021)). M2 macrophages were polarized using 25 ng/mL of IL-4 (StemCell Technologies, #78045; https://www.stemcell.com/ (Accessed on 9 September 2021)) and IL-13 (Stemcell Technologies, #78029; https://www.stemcell.com/ (Accessed on 9 September 2021)). Polarization lasted 48 h at which time cells were treated with sTREM2, as described above, and then washed and lysed with TRIzol and RNA isolated for RT-qPCR. The supernatants were collected and stored at −80 °C for later experiments.

### 2.4. Reverse Transcription-Quantitative Polymerase Chain Reaction (RT-qPCR)

Total RNA was isolated from THP-1 monocytes or macrophages using 100 µL TRIzol reagent (Invitrogen) following the manufacturer’s instructions. For THP-1 monocytes, cells in suspension were removed from the plate and centrifuged at 300× *g* for 5 min, supernatant was removed and stored at −80 °C for future experiments, and TRIzol reagent was added to the cell pellet. For macrophages, due to their adherence, supernatant was removed from the plate and stored at −80 °C for future experiments, and TRIzol reagent was added directly to the wells. The manufacturer’s protocol was followed except that all centrifugations were carried out at 14,000× *g* with the addition of an extra chloroform wash step, which included diluting the fractionated aqueous layer to 500 μL in dimethyl pyrocarbonate (DEPC)-treated dH_2_O and fractionating another aqueous layer using equal parts chloroform and solution. The separated aqueous layer volume was then precipitated using 450 μL isopropanol and 45 μL 3 M sodium acetate (pH 5.2) for 30 min at room temperature. The mixture was centrifuged at 14,000× *g* for 5 min, the supernatant was decanted, and the RNA pellet was washed three times with ice-cold 75% ethanol and solubilized in 15 μL DEPC-treated dH_2_O at 55 °C for 10 min. RNA was quantified using the ThermoFisher Scientific NanoDrop spectrophotometer and was then either used directly for reverse transcription or stored at −80 °C for later use.

Reverse transcription (cDNA synthesis) was performed using an M-MLV reverse transcription kit 200 U/μL (Invitrogen) supplied with 2.5 μM dNTP mix, 5× first strand buffer, and 0.1 M DTT (*Dithiothreitol*). Total RNA (100 ng) was mixed with 200 ng random hexamer primer (Invitrogen, #SO142; Burlington, ON, Canada) (https://www.thermofisher.com/ca/en/home/brands/invitrogen.html (Accessed on 9 September 2020)), 0.5 μM dNTP, and DEPC-treated dH_2_O, heated at 65 °C for 5 min, and then directly placed on ice. Samples were centrifuged and then combined with 1X first strand buffer and 10 mM DTT and heated at 37 °C for 2 min. Reverse transcriptase (200 U M-MLV) was finally added to yield a 20 μL reaction and was allowed to incubate at room temperature for 10 min before incubating at 37 °C for 50 min. The reaction was inactivated at 70 °C for 15 min. The complementary DNA (cDNA) was diluted 10-fold for use in subsequent qPCR reaction.

qPCR amplification was performed in a final volume of 10 µL using PowerTrack SYBR Green Master Mix (ThermoFisher, #A46012; Ottawa, ON, Canada) (https://www.thermofisher.com/ca/en/home.html (Accessed on 9 September 2020)) following the manufacturer’s cycling protocol for 45 cycles. Samples were run in duplicates, and the optimal annealing temperature for each primer was determined using a gradient style assay and using the annealing temperature that yielded the best amplification curve. The expression of several cytokines was assessed using specific primers for TNF-α, IL-1β, IL-10, TARC/CCL17, or/and IL-6, and housekeeping gene β-actin (Supplementary Table S1). Relative quantification using the delta Ct method outlined by Ganger et al. in 2017 was used to quantify gene expression normalized with β-actin.

### 2.5. Enzyme-Linked Immunosorbent Assay (ELISA)

An Anogen Multiplex Human Cytokine ELISA Kit (#EM10001, Anogen-Yes Biotech Laboratories Ltd., Mississauga, ON, Canada) was used in the semi-quantitative determination of pro-inflammatory cytokine expression in polarized M0, M1, and M2 macrophages being treated by sTREM2. The pro-inflammatory cytokines analyzed included IL-1α, IL-1β, IL-6, IL-8, IFN-γ, GM-CSF, MCAF, and TNF-α. Standards were diluted in RPMI1640 supplemented with FBS. Two concentrations of each standard were used to generate standard curves, including a high concentration provided by the manufacturer and a 1:32 dilution. The manufacturer’s protocol was followed. Each of the samples were tested in duplicate. A Synergy HTX microplate reader was used to read the plates. A RayBio human TREM2 ELISA kit (Cat# ELH-TREM2m, RayBiotech, Peachtree Corners, GA, USA) was used to detect and quantify sTREM2 in culture media of M0, M1, and M2 macrophages at 24 and 48 h following the manufacturer’s protocol.

### 2.6. Statistical Analysis

Data are presented as the Mean ± SD, and each experiment was repeated at least 3 times. Statistical analysis for single comparison was performed using a Student’s *t*-test. For multiple comparisons, a one-way ANOVA was performed. The criterion for statistical significance was *p* < 0.05. All analyses were carried out using GraphPad Prism 10 software.

## 3. Results

### 3.1. sTREM2-Induced Expression of Cytokines in THP-1 Cells

**Figure 1:**
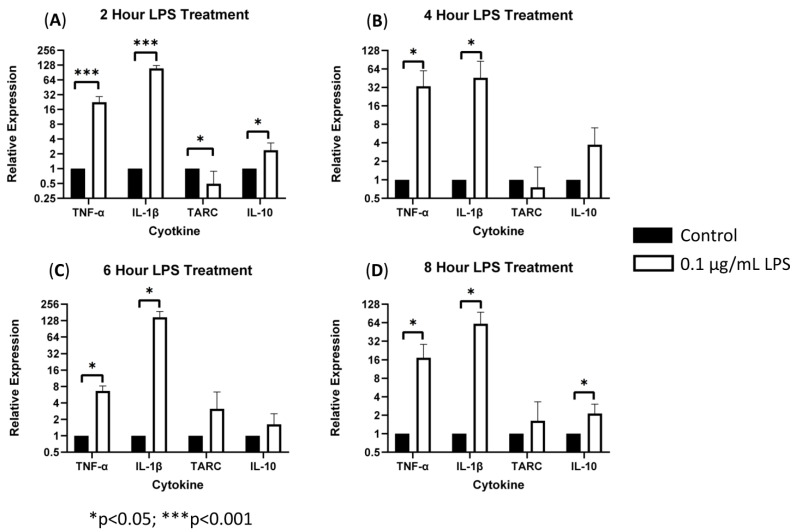
LPS-induced cytokine expression in THP-1 cell model: treating THP-1 monocytes with 0.1 μg/mL LPS strongly stimulated expression of TNF-α and IL-1β (*** = *p* < 0.001; * = *p* < 0.05) as well as TARC and IL-10 expression (* *p* = 0.05) as compared to negative controls at 2 (**A**), 4 (**B**), 6 (**C**), and 8 (**D**) hours post treatment.

To characterize the role of sTREM2 in inducing cytokine expression, THP-1 monocytes were treated with 0.1 μg/mL or 1.0 μg/mL recombinant human sTREM2, and cytokine expression (IL-1β, TNF-α, IL-10, and TARC/CCL17) was assessed at 2, 4, 6, and 8 h post treatment using RT-qPCR. Negative control cells were treated with an equal volume of sterile H_2_O. Bacterial LPS was used as a positive control to exemplify induction of an inflammatory response in this model since it is known to strongly stimulate expression of inflammatory cytokines in monocytes. LPS at 0.1 μg/mL strongly stimulated the expression of TNF-α and IL-1β and to a lesser extent TARC/CCL17 and IL-10 at 2, 4, 6, and 8 h post treatment (Figure 1). Overall, LPS served as a strong positive control to ensure functionality of the primers and induction of inflammatory cytokine expression.

**Figure 2:**
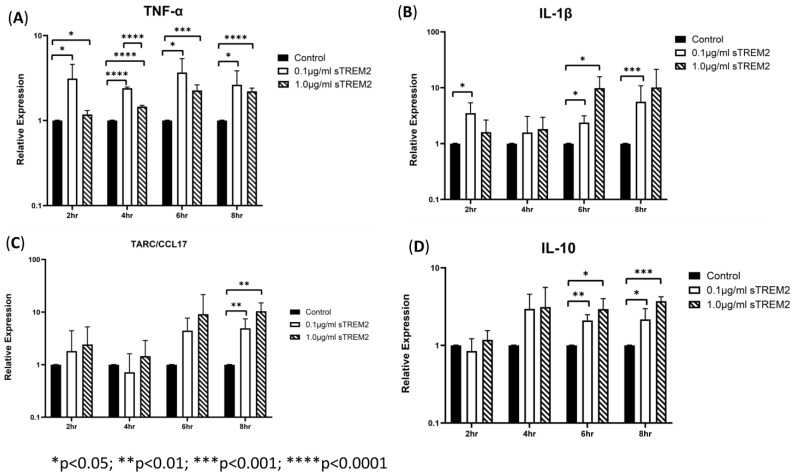
sTREM2-induced cytokine expression in THP-1 cell model: treating THP-1 cells with 0.1 μg/mL or 1.0 μg/mL sTREM2 induced the expression of (**A**) TNF-α, (**B**) IL-1β, (**C**) TARC, and (**D**) IL-10. Data presented as Mean + SD.

THP-1 cells were then treated with 0.1 μg/mL or 1 µg/mL sTREM2 for 2 h, 4 h, 6 h, and 8 h, and the negative controls were treated with the same volume of sterile water used to resuspend sTREM2. At 2 h of treatment, sTREM2 stimulated the expression of IL-1β (* *p* < 0.03) and TNF-α (* *p* < 0.05) but failed to stimulate expression of TARC or IL-10 compared to negative controls (Figure 2). At 4 h of treatment, the expression of cytokines remained like the 2 h treatment effects; where TNF-α (**** *p* < 0.0001) was increased, and both IL-1β and IL-10 were also induced but not to a statistically significant level. TARC expression remained similar to control levels. At 6 h of treatment (Figure 2), both concentrations (0.1 μg/mL and 1.0 μg/mL) of sTREM2 were able to significantly stimulate the expression of TNF-α, IL-1β, and IL-10 compared to control THP-1 cells. At 0.1 μg/mL, sTREM2 induced the expression of TNF-α (* *p* < 0.05) and IL-1β (*p < 0.05) to the levels consistent with what has been previously seen; and IL-10 (** *p* < 0.01) expression was significantly induced. TARC was also induced but not to a statistically significant degree. A similar pattern is seen following treatment using 1.0µg/mL sTREM2 where TNF-α (*** *p* < 0.001), IL-1β (* *p* < 0.05), and IL-10 (* *p* < 0.05) were increased as compared to controls. At this time point, expression levels for each TNF-α, IL-1β, and IL-10 expression levels were at the highest and most consistent levels following both 0.1 μg/mL and 1.0 μg/mL sTREM2 treatment compared to either of the other time points. Consistent with the results shown at previous time points, at 8 h of treatment (Figure 2), 0.1 µg/mL sTREM2 was able to increase the expression of TNF-α (* *p* < 0.05), IL-1β (*** *p* < 0.001), TARC (** *p* < 0.01), and IL-10 (*p < 0.05). At the concentration of 1.0 μg/mL, sTREM2 was able to exert similar or stronger effects on THP-1 cells as 0.1 μg/mL sTREM2 with increased expression of TNF-α (**** *p* < 0.0001) and IL-10 (*** *p* < 0.001).

Together these results show that 0.1 μg/mL sTREM2 can consistently stimulate the expression of major cytokines TNF-α, IL-1β, TARC, and IL-10 with the expression of inflammatory cytokines TNF-α and IL-1β throughout the time course (except IL-1β at 4 h), but it can also induce the expression of anti-inflammatory cytokines TARC and IL-10 at later time points. On the other hand, 1.0 μg/mL sTREM2 was also capable of stimulating expression of these cytokines. However, it is interesting that large amounts of sTREM2 does not exponentially increase cytokine production or induce cell death as measured by cell viability assay (cell viability was within 95%). sTREM2 can induce the expression of these cytokines in THP-1 but it is not currently known what receptor sTREM2 binds with on the membrane of THP-1 cells. This prompted us to investigate the mechanisms behind sTREM2-induced cytokine expression. To further investigate, a commercially available TREM2 antibody was employed in an attempt to try to neutralize sTREM2′s ability to induce expression of cytokines in THP-1 cells.

### 3.2. TREM2 Monoclonal Antibody (mAb) Partially Alleviated sTREM2-Induced Expression of Cytokines

A commercially available rat anti-human IgG_2B_ TREM2 antibody (R&D Systems) was used to assess the antibody’s ability to bind and neutralize sTREM2. FACS analysis showed that this TREM2 antibody binds to TREM2 on THP-1 cells, but the isotype control antibody does not; while SPR (surface plasmon resonance) analysis confirmed that the TREM2 antibody binds to recombinant sTREM2 (Supplementary Figures S1 and S2). The recombinant sTREM2 and the antibody were then co-incubated at equal concentrations of 0.1 μg/mL for 30 min at 37 °C prior to treating THP-1 cells and then added to the cells for different time points. Cells were harvested at 2, 4, 6, and 8 h post treatment, and cytokine expression was assessed by RT-qPCR.

**Figure 3:**
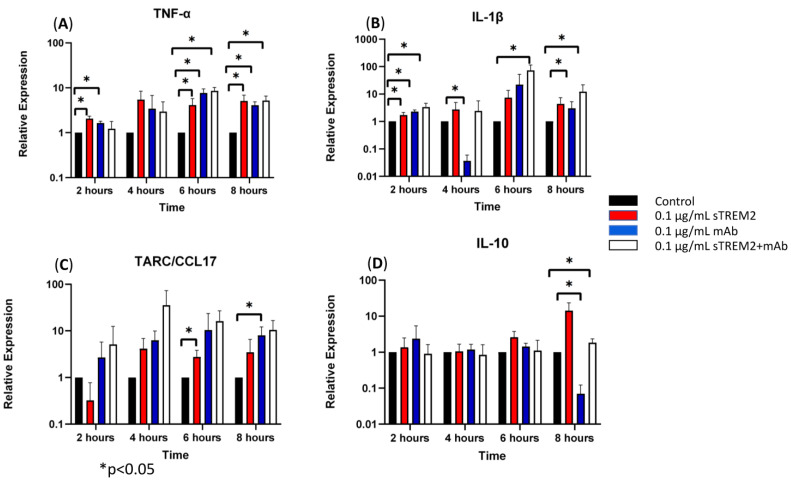
The effects of a TREM2 antibody (mAb) on sTREM2-induced cytokine expression: THP-1 cells were treated with 0.1 μg/mL sTREM2, mAb, or both sTREM2+mAb for 2, 4, 6, and 8 h, and cytokine expression of (**A**) TNF-α, (**B**) IL-1β, (**C**) TARC, and (**D**) IL-10 was assessed using qPCR. Data presented as Mean + SD.

At 2 h of treatment, 0.1 μg/mL of sTREM2 was capable of stimulating the expression of inflammatory cytokines in THP-1 cells as previously shown (Figure 3). The addition of only the anti-TREM2 mAb to THP-1 cells increased the expression of TNF-α (* *p* < 0.05) and IL-1β (* *p* < 0.05) (Figure 3). This may likely be attributed to the antibody engaging the TREM2 receptor on the cell membrane, which has been shown to play roles in activation and similar pathways [13,19]. The addition of 0.1 μg/mL sTREM2 pre-incubated with 0.1 μg/mL TREM2 mAb showed reduced expression of TNF-α and IL-10 as compared to 0.1 μg/mL sTREM2 alone or mAb alone; however, it is interesting that sTREM2+mAb increased the expression of IL-1β (* *p* < 0.05) to levels higher than either sTREM2 or mAb alone (Figure 3). This may be, as a result, recognized as a complex of sTREM2/mAb being internalized or bound to unknown receptor(s) on the cell membrane, which may trigger an alternative reaction to induce expression of the cytokine. At 4 h of treatment (Figure 3), TREM2 mAb alone showed a significant decrease in IL-1β expression (* *p* < 0.05). sTREM2+mAb stimulated the expression of TARC as compared to sTREM2-treated cells, but it was not significant due to a large variation (Figure 3). At 6 h of treatment (Figure 3), THP-1 cells treated with 0.1 μg/mL sTREM2 showed increased expression of TNF-α (* *p* < 0.05) and TARC (* *p* < 0.05). Treatment with TREM2 mAb alone increased the expression of TNF-α. Interestingly, treatment with sTREM2 + mAb increased the expression of TNF-α and IL-1β (* *p* < 0.05) as compared to the control but not to the cells treated with 0.1 µg/mL sTREM2 (Figure 3). At 8 h of treatment, sTREM2 at 0.1 μg/mL induced the expression of TNF-α (* *p* < 0.05), IL-1β, TARC, and IL-10 as compared to control THP-1 cells. Treatment with mAb also stimulated the expression of TNF-α (* *p* < 0.05) and TARC (* *p* < 0.05) as compared to control cells but significantly decreased the expression of IL-10 (* *p* < 0.05) as compared to control THP-1 cells. sTREM2+mAb treatment increased the expression of IL-1β (* *p* < 0.05) while decreasing the expression of IL-10 (* *p* < 0.05) as compared to sTREM2-treated cells. Interestingly, sTREM2+mAb treatment had little effect on TNF-α expression as compared to sTREM2-treated cells.

**Figure 4:**
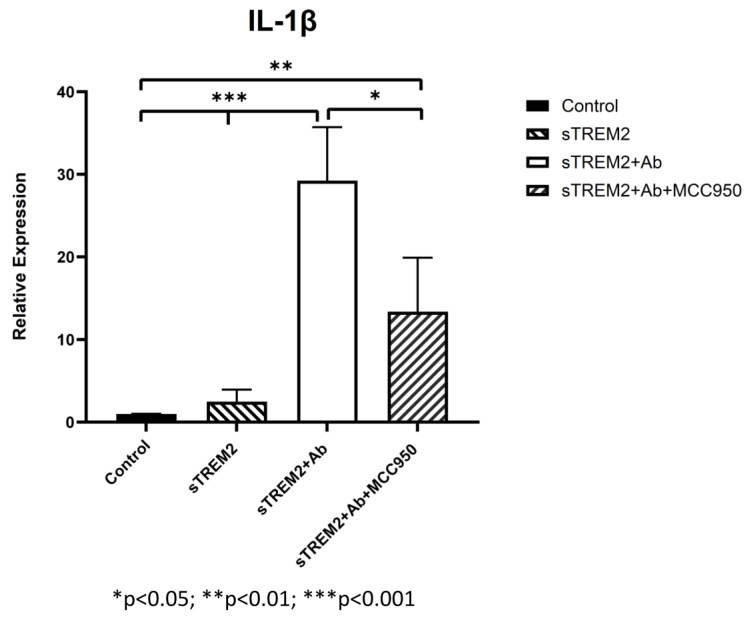
Inhibition of NLRP-3 inflammasome-mediated release of IL-1β induced by the complex of sTREM2+mAb. THP-1 cells were treated for 6 h with 0.1 μg/mL sTREM2, TREM2 antibody (mAb), or 0.1 μg/mL sTREM2+mAb (a monoclonal TREM2 antibody) and 0.1 μg/mL sTREM2+mAb with a selective NLRP3 inhibitor MCC950 (7.5 nM). Data presented as Mean + SD. * *p* < 0.05; ** *p* < 0.01; *** *p* < 0.001.

It is noted that the complex of sTREM2+mAb strongly stimulated expression of IL-1β (Figure 3B); however, the mechanism for which this occurred is unknown. It is likely that sTREM2+mAB may be recognized as an immune complex which stimulates the activation of the NLRP3 inflammasome to release IL-1β. To test that, an NLRP3 inhibitor MCC950 was used to target NLRP3 activation. THP-1 cells were treated for 6 h with 0.1 μg/mL sTREM2, 0.1 μg/mL sTREM2+mAb, and 0.1 μg/mL sTREM2+mAb with MCC950 at 7.5 nM. qPCR results show that MCC950 was able to partially inhibit expression of IL-1β (Figure 4). This indicates that the NLRP3 inflammasome is involved in the sTREM2/mAb complex-induced release of IL-1β in THP-1 cells.

The above results reinforce the theory that sTREM2 is capable of stimulating cytokine expression in this THP-1 cell model. Moreover, it was shown that treatment of the cells with sTREM2+mAb complex was capable of alleviating the expression of some cytokines including TNF-α and IL-10 to certain degree at some time points but significantly induced the expression of other cytokine such as IL-1β as compared to control THP-1 cells or sTREM2-treated cells. Treatment of THP-1 cells with only mAb also upregulated the expression of TNF-α and IL-1β at 2 h, 6 h, or 8 h as compared to control cells or to sTREM2 treatment alone. Since this is an anti-TREM2 mAb, the expression of these cytokines mediated by this mAb treatment was likely due to the engagement of the cell membrane TREM2 receptor. Together these results suggest that targeting either sTREM2 or TREM2 may elicit a cellular response. Here it is important to note that while the mAb incubated with sTREM2 reduced the expression of some cytokines, they were not reduced to levels equal to or less than the controls but did reduced expression as compared to sTREM2-treated cells.

### 3.3. sTREM2 Induced Expression of Cytokines in Macrophages Differentiated and Polarized from THP-1 Cells

It is known that TREM2 functions primarily in macrophages and microglia in the innate immune response [7]. To test whether sTREM2 also induces the expression of cytokines in macrophage-like cells, THP-1 cells were first differentiated into macrophages (M0) and then polarized to the M1 or M2 phenotype as described in the Section 2.3. The protocol for THP-1 cell differentiation and polarization to M0, M1, and M2 macrophages has been validated by FACS analyses of marker expressions on these cells (Supplementary Figures S3 and S4). The M0, M1, and M2 cells were washed three times with fresh culture media and then treated with either 0.1 μg/mL or 1.0 μg/mL sTREM2 for 2 h and 8 h, and control M0, M1, and M2 cells were treated with sterile water. Following treatment, cytokine expression was quantified by RT-qPCR. Protein expression of cytokines was analyzed using a human cytokine multiplex ELISA kit.

**Figure 5:**
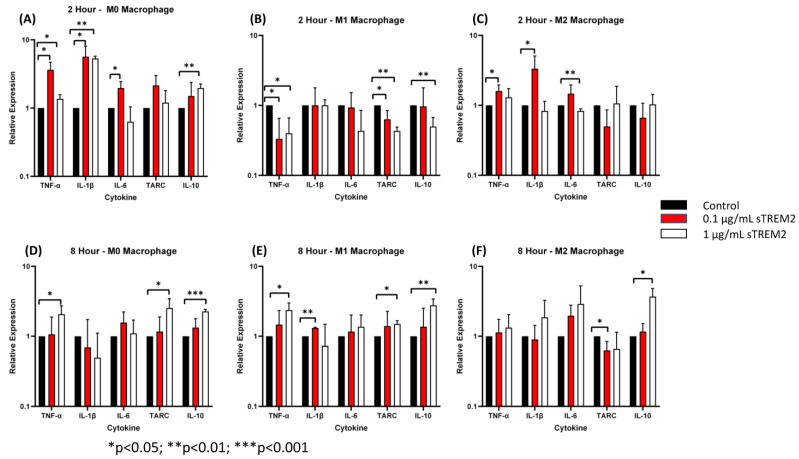
The effects of sTREM2 on cytokine expression in THP-1-derived macrophages: (**A**). Cytokine expression in M0 macrophages following 2 h of sTREM2 treatment. (**B**). Cytokine expression in M1 macrophages following 2 h of sTREM2 treatment. (**C**). Cytokine expression in M2 macrophages following 2 h of sTREM2 treatment. (**D**). Cytokine expression in M0 macrophages following 8 h of sTREM2 treatment. (**E**). Cytokine expression in M1 macrophages following 8 h of sTREM2 treatment. (**F**). Cytokine expression in M2 macrophages following 8 h of sTREM2 treatment. Data presented as Mean + SD.

At 2 h of treatment of M0 cells (Figure 5A), 0.1 μg/mL sTREM2 induced the largest expressional changes in TNF-α (*p* < 0.001) and IL-1β (*p* < 0.001) but also stimulated the expression of IL-6 (*p* = 0.02). sTREM2 at 1.0 μg/mL was able to stimulate the expression of TNF-α and IL-1β (*p* = 0.03) as well as IL-10 (*p* = 0.004) but not in stimulating IL-6 or TARC as compared to untreated M0 macrophages (Figure 5A). Generally speaking, 1 µg/mL sTREM2 was not as strong as 0.1 µg/mL sTREM2 in inducing cytokine expression except with IL-10 (Figure 5).

Following a 2 h treatment with 0.1 μg/mL sTREM2, M1 macrophages showed a reduction in TNF-α expression (*p* < 0.05) and TARC (*p* < 0.05) compared to the control (Figure 5B). This was also true for 1.0 μg/mL sTREM2-treated M1 macrophages showing decreases in the expression of TNF-α (*p* = 0.02), TARC (*p* < 0.01), and IL-10 (*p* < 0.01). This supports the notion that sTREM2 may play a role in initiating inflammation rather than promoting it in M1 cells that are already “programmed” to be an inflammatory phenotype. This may also be supported by the fact that 1.0 μg/mL sTREM2 shows reduced expression of cytokines and, therefore, the accumulation of sTREM2 may result in a reduction in cytokine expression or initiate a different response.

In M2 macrophages (Figure 5C), at 2 h of treatment, 1.0 μg/mL sTREM2 had minimal effects on cytokine expression with levels remaining similar to that of control M2 macrophages except for IL-6 (*p* < 0.01) which was decreased as compared to the control and 0.1 µg/mL sTREM2-treated cells. Treatment of M2 macrophages with 0.1 μg/mL sTREM2 stimulated the expression of TNF-α (*p* < 0.05) (Figure 5C). In M0 and M2 macrophages, 0.1 μg/mL sTREM2 primarily exerted its effects on pro-inflammatory cytokines, supporting the notion that sTREM2 likely plays a role in initiating the inflammatory response in cells at an early stage. On the contrary, 1.0 μg/mL sTREM2 was relatively less efficient at stimulating expression of cytokines in M0, M1, or M2 macrophages.

At 8 h post treatment the effects of 1.0 μg/mL sTREM2 had much more of an effect on the expression of cytokines in all M0, M1, and M2 macrophages (Figure 5D–F) as compared to the effects at 2 h post treatment. Interestingly, the effects of 0.1 μg/mL sTREM2 on cytokine expression in M0, M1, and M2 cells at 8 h was reduced as compared to the effects on cytokine expression at 2 h post treatment. This may indicate that sTREM2s effects are relatively quick acting and short. In M0 macrophages following treatment with 1 μg/mL sTREM2, the expressions of TNF-α (* *p* < 0.05), TARC (* *p* < 0.05), and IL-10 (** *p* < 0.01) were induced in comparison to the control M0 macrophages at 8 h post treatment (Figure 5D). It is noted that at 8 h, the expression of anti-inflammatory cytokines TARC and IL-10 was significantly upregulated, indicating that at higher concentrations and a longer exposure to sTREM2, M0 macrophages expressed high levels of anti-inflammatory cytokines as compared to control M0 macrophages.

In M1 cells at the 8 h time point (Figure 5E), treatment with 0.1 μg/mL sTREM2 was able to stimulate the expression of IL-1β (*p* < 0.001) as compared to untreated M1 macrophages; while treatment with 1.0 μg/mL sTREM2 stimulated the expression of TNF-α (*p* = 0.02), TARC (*p* = 0.007), and IL-10 (*p* = 0.008) as compared to control M1 macrophages (Figure 5E). It is important to note here that M1 macrophages are polarized towards a pro-inflammatory state, and therefore for sTREM2 to strongly induce the expression of TARC and IL-10 at 8 h in this cell type supports the idea that prolonged exposure to sTREM2 may initiate anti-inflammatory response in M1 macrophages. In M2 macrophages (Figure 5F), 1.0 μg/mL sTREM2 also strongly stimulated the expression of IL-10 (*p* = 0.01) in comparison to control M2 macrophages, which coincide with what we have previously presented earlier, but TARC expression was decreased in M2 macrophages treated with sTREM2. The expression of IL-1β and IL-6 was also increased following treatment, although this was not shown to be statistically significant due to variations. sTREM2 at 0.1 μg/mL showed minimal effects on cytokine expression in M2 macrophages at the 8 h time point compared to control macrophages, except reduced TARC expression.

In the context of our model at early (2 h) time points, sTREM2 was capable of stimulating the expression of pro-inflammatory cytokines TNF-α and IL-1β and to a lesser extent IL-6 in M0 and M2 macrophages but reducing the expression of TNF-α in M1 macrophages at 2 h. At the later time point (8 h), a higher concentration of sTREM2 was able to stimulate the expression of TNF-α or/and IL-1β in M0 and M1 macrophages as well as anti-inflammatory cytokines IL-10 or/and TARC in M0, M1, or M2 macrophages; however, it reduced TARC in M2 macrophages. The findings in M0 macrophages are essentially the same as observed in THP-1 cells.

To test whether the elevated expression of pro-inflammatory cytokines was translated at the protein level in treated M0, M1, and M2 (Table 1) macrophages, a human cytokine multiplex kit was used to detect the levels of IL-1α, IL-1β, IL-6, IL-8, GM-CSF, IFN-γ, MCAF (monocyte chemotactic and activating factor), and TNF-α using the supernatants from controls and cells treated with 0.1 μg/mL or 1.0 μg/mL sTREM2 at 2 h (Table 1) (for standard curves, please see Supplementary Figure S5). Like previously shown results (Figure 5), M0 macrophages treated with 0.1 μg/mL sTREM2 increased the expression of IL-1β and TNF-α as compared to the control. TNF-α protein expression in M0 macrophages treated with 0.1 μg/mL sTREM2 showed ~12 pg/mL increase in protein level as compared to untreated M0 macrophages. Like qPCR results in Figure 5A, 1.0 μg/mL sTREM2 treatment increased IL-1β by about 9 pg/mL. The only other cytokine that seemed to be increased by 0.1 μg/mL sTREM2 treatment in M0 macrophages compared to controls seemed to be IFN-γ, which showed approximately 14 pg/mL. Treatment with sTREM2 at 1.0 μg/mL also increased the protein expression of IL-1β in M0 cells by 8 pg/mL. In M1 macrophages, qPCR showed that treatment with sTREM2 either at 0.1 or 1 µg/mL reduced the expression of TNF-α, TARC, and IL-10 (Figure 5B); while ELISA showed slightly reduced TNF-α levels in treated M1 cells as compared to untreated M1 macrophages (Table 1).

In M2 macrophages (Table 1), treatment with sTREM2 at 2 h showed a slightly increased expression of TNF-α or IL-1β, showing the same trend as RT-qPCR results in M2 cells at 2 h (Figure 5C). Interestingly, an increase in IFN-γ protein expression was also seen in 0.1 μg/mL sTREM2-treated M2 macrophages. However, treatment of M2 cells with 1.0 μg/mL sTREM2 increased the protein level of IL-1β (Table 1) by ~9.3 pg/mL. The protein levels of IL-8, IFN-γ, and GM-CSF in M2 cells treated with 1 µg/mL sTREM2 showed increases of 18 pg/mL, 35 pg/mL, and 32 pg/mL, respectively, as compared to untreated M2 cells. These results support the notion that sTREM2 is a key player in activating cells and initiating cytokine expression.

Together the increased expression of pro-inflammatory cytokines in mainly M0 and M2 macrophages both at the transcriptional and protein levels shows that sTREM2 can induce cytokine expression in myeloid-derived cells. Interestingly, sTREM2 decreased the expression of cytokines in M1 macrophages at the early time point but increased the expression of the cytokines at the later time point. This supports the notion that M1 cells have already been programed to be in a pro-inflammatory state (M1 phenotype) and that sTREM2 does not have a role in further activating M1 cells towards a pro-inflammatory state. However, sTREM2 was capable of stimulating the expression of pro-inflammatory cytokines at the early time point in M0 and M2 cells and TNF-α in M0 cells/both TNF-α and IL-1β in M1 cells at the later time point. More importantly, sTREM2 induced the expression of anti-inflammatory cytokines at the later time point in M0, M1, and M2 cells except for TARC in M2 cells at 8 h. These observations are essentially similar to what was seen in THP-1 monocytes except M1 cells at the early time point.

### 3.4. Inhibition of JNK Blocks sTREM2-Induced Expression of Cytokines in THP-1 Cells

It remains unknown which pathways sTREM2 may use to induce the expression of cytokines in THP-1 monocytes and macrophages. Several chemical inhibitors were employed in the assays similar to previous sTREM2 treatments. MG-132 was used to inhibit proteasome and, therefore, NFκB activation. SP600125 was used to inhibit JNK, which is downstream in the MAPK signaling pathway. Piceatannol was chosen as a Syk inhibitor, which is the major kinase for TREM2 signaling activation. While the IC50′s of the inhibitors was provided by the manufacturer and subsequently used in the following experiments, it was not known whether inhibitors would cause cell apoptosis or death in THP-1 cells. In order to explore this possibility, a cell viability test was completed to assess for cell loss over a period longer than the intended treatment time with the IC50s of each inhibitor indicated by the manufacturer. THP-1 cells were counted and plated at a concentration of 500,000 cells/mL and treated with an inhibitor. At 8 h, cells were collected and recounted, which all remained within 95% viability. THP-1 cells were pre-incubated with each inhibitor for 15 min at 37 °C to ensure full diffusion of the inhibitor prior to the administration of 0.1 μg/mL sTREM2. Cytokine gene expression was assessed by RT-qPCR at the 6 h time point to reduce sample size as this time point showed consistent sTREM2-induced cytokine expression in our model.

**Figure 6:**
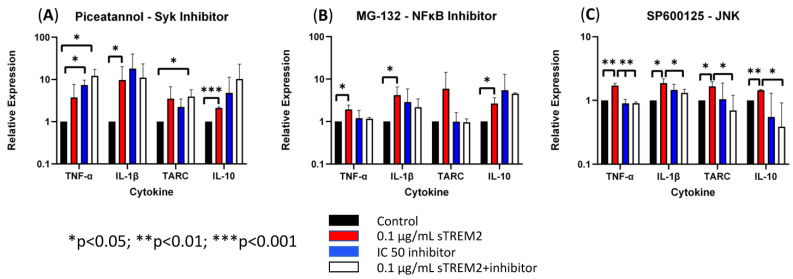
The effects of Syk, NFκB, and JNK inhibitors on sTREM2-induced cytokine expression (**A**). The effect of TREM2 inhibitor piceatannol (15 μM) on cytokine expression; (**B**). The effect of MG-132 (100 nM) on cytokine expression; (**C**). The effect of JNK inhibitor SP600125 (90 nM) on cytokine. Data presented as Mean + SD.

First, Syk was targeted by using the inhibitor Piceatannol to check whether sTREM2-induced cytokine expression in THP-1 monocytes can be inhibited. Under normal physiological conditions TREM2 signaling is shown to recruit and phosphorylate Syk enzyme to communicate downstream after ligand binding and association with its adaptor protein DAP12 [2,7,10]. Piceatannol was used to treat THP-1 cells at an IC50 of 15 μM (Figure 6A). Treatment with 0.1 μg/mL sTREM2 still induced the expression of TNF-α, IL-1β, TARC, and IL-10 as compared to untreated cells. While treatment with 15 μM piceatannol did not induce cell death in the previous cell viability experiment, the inhibitor did not affect sTREM2-induced expression of cytokines. However, it appeared that the inhibitor slightly enhanced the expression of these cytokines as compared to both sTREM2-treated and untreated THP-1 cells. It is important to note that this experiment did not show that sTREM2 uses the TREM2–Syk signaling pathway to induce cytokine expression.

Next, the proteasome inhibitor MG-132 was used to test whether NFκB signaling was involved in sTREM2-induced cytokine expression (Figure 6B). MG-132 was used to treat THP-1 cells at an IC50 of 100 nM as indicated by the manufacturer. It was seen that 0.1 μg/mL of sTREM2 consistently stimulated expression of TNF-α, IL-1β, TARC, and IL-10 as shown previously. THP-1 cells treated with 100 nm of MG-132 showed slightly increased expression in IL-1β and IL-10, but TNF-α and TARC expression remained relatively unchanged by 100 nM of MG-132. Interestingly, 100 nM of MG-132 appeared to have some effect on sTREM2-induced cytokine expression in THP-1 cells but did not completely inhibit the response. TNF-α and TARC expression induced by sTREM2 treatment was reduced to levels similar to untreated control cells, but IL-1β and IL-10 expression was only slightly reduced. Although those were not statistically significant, a trend is visible. The fact that MG-132 did not completely abolish sTREM2-induced expression of IL-1β and IL-10 supports the complexity of sTREM2 and its ability to differentially regulate cytokine expression in this cell model.

Lastly, JNK was targeted by the inhibitor SP600125. JNK is a kinase downstream of MAPK signaling, a major pathway for regulating cytokine expression. Our previous studies showed that JNK is activated by beta-amyloid peptide [20,21]. In this study, SP600125 was used at an IC50 of 90 nM to inhibit JNK1, JNK2, and JNK3 as indicated by the manufacturer. Consistent with the previous assay, sTREM2 induced the expression of cytokines in THP-1 cells (Figure 6C). The addition of only the inhibitor did not alter cytokine expression. It was observed that after a short incubation with SP600125 and following the addition of sTREM2, the expression of cytokines was nearly completely abolished by the inhibitor (Figure 6C). It was primarily seen that the expression of TNF-α (** *p* = 0.01), TARC (* *p* < 0.05), and IL-10 (** *p* < 0.01) was inhibited to levels equal to or lower than the untreated control samples. IL-1β expression, however, was not completely reduced to the control level, but it was partially inhibited as compared to sTREM2-treated cells. Given the fact that SP600125, a well-documented JNK inhibitor [20,21], was able to inhibit the expression of inflammatory cytokines nearly completely in THP-1 cells induced by 0.1 μg/mL sTREM2, it indicates that MAPK–JNK may be the primary cellular pathway used by sTREM2 to induce the expression of these cytokines in THP-1 cells. The one-way ANOVA showed a strong statistically significant difference between the treated and control groups (** *p* = 0.01), which further supports the notion that sTREM2 may primarily use the MAPK–JNK signaling pathway to up-regulate the expression of cytokines in THP-1 cells.

### 3.5. Polarized Macrophages Differentially Shed sTREM2

While it is widely known that TREM2 is expressed on myeloid-derived cells like macrophages and microglia [7], studies have found that M2 macrophages express larger quantities of TREM2 than either M0 or M1 cells [22], but whether TREM2 on the cell membrane can be shed into culture media has yet to be definitively confirmed in our THP-1/macrophage model. To figure out whether differentiating macrophages shed TREM2 into culture media, macrophages were differentiated from THP-1 cells as described in the Section 2.3, and at 24 and 48 h during polarization, supernatants from polarized M0, M1, and M2 macrophages were collected and stored at −80 °C. Later, a human TREM2 ELISA kit was used to quantify the levels of sTREM2 in culture supernatant (for standard curve of the assay, please see Supplementary Figure S6).

**Figure 7:**
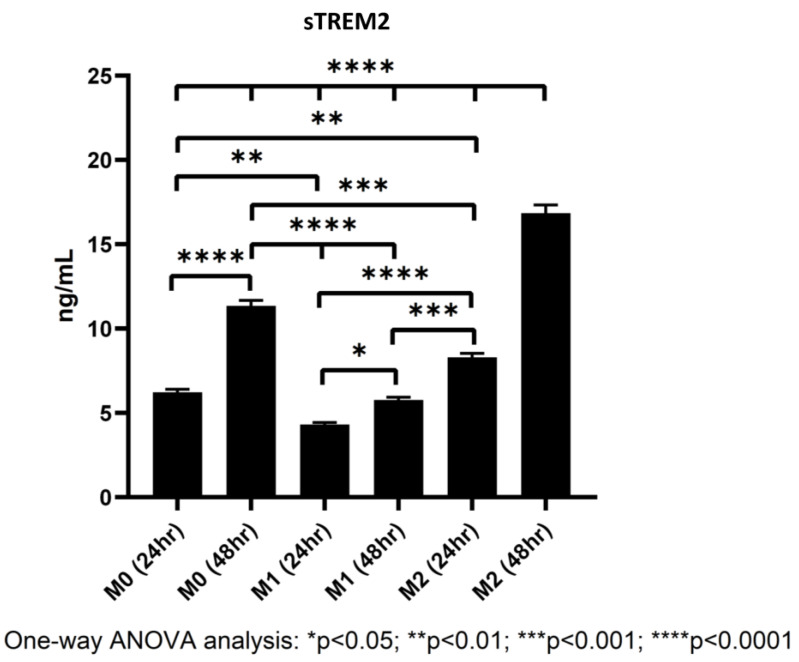
Levels of sTREM2 in the culture media of M0, M1, and M2 macrophages at 24 h and 48 h detected by ELISA.

In all samples, sTREM2 was readily detectable under current model conditions in cell culture media of polarized M0, M1, and M2 macrophages. It was showed that M2 macrophages shed sTREM2 to the highest amount followed by M0 and M1 macrophages. M2 macrophages shed nearly double the amount of sTREM2 at 24 h as compared to M1 macrophages and over triple the amount at 48 h (Figure 7). The levels of sTREM2 increased approximately twofold between 24 h and 48 h in M0 and M2 macrophages but not in M1 macrophages. In M0 macrophages at 24 h, 6.23 ng/mL sTREM2 was detected as compared to around 11.35 ng/mL at 48 h. In contrast, sTREM2 level in culture media of M1 macrophages was 4.31 ng/mL at 24 h but 5.77 ng/mL at 48 h, which was only increased by about 1.5 ng/mL at 48 h (Figure 7). M2 cells shed the highest levels, reaching around double that of M1 at 24 h with 8 ng/mL detectable in culture media and 16.85 ng/mL at 48 h. It is important to note that the levels of sTREM2 in culture media for M2 macrophages at 48 h was likely to be slightly overestimated due to the fact that it was outside the range of concentration generated by the standard curve (Supplementary Figure S6). These results clearly indicate that M2 macrophages shed the largest amount of sTREM2 in culture media, followed by M0 and then M1 macrophages.

## 4. Discussion

### 4.1. sTREM2-Induced Cytokine Expression in THP-1 Monocytes

In recent years, the roles of sTREM2 in microglial function [14] and in AD are becoming increasingly apparent. Importantly, sTREM2 has been shown to be increased in CSF, with variable levels dependent on the variants, just before the clinical onset of symptoms in AD [17,18]. Microglia are considered as resident macrophages in the central nervous system (CNS). Studies show that microglia are derived from macrophages during CNS development, and macrophages can also migrate into the CNS as well [23]. The goal of this study was to determine whether sTREM2 could induce an inflammatory response in a myeloid-derived cell model. The THP-1 cell line is a human monocytic leukemia line of myeloid origin and is used as a model in this study. The cells were treated with different concentrations of sTREM2, and the cytokine expression and relevant signaling pathways involved were investigated. It was shown that sTREM2 can induce the expression of several pro-inflammatory and anti-inflammatory cytokines in this THP-1 model at different time points, suggesting that sTREM2 can induce transcriptional changes in these cells. This could indicate that sTREM2 plays crucial roles in innate immune response and suggest that sTREM2 has a strong regulatory function, likely involved in process of innate immune and inflammatory responses. This is also supported by the fact that sTREM2 is detectable in both CSF and plasma. sTREM2 levels detectable in the plasma of AD patients, non-AD individuals, and patients with other neurodegenerative diseases has been reported to range anywhere from 10 to 50 ng/mL [24] and is significantly lower in CSF, at around 10 ng/mL, with cognitively impaired individuals typically displaying slightly higher levels in CSF compared to unimpaired individuals [25].

The concentrations of sTREM2 used in this study (0.1 and 1.0 µg/mL) were relatively higher than the physiological levels detected in plasma and CSF but still encapsulated that sTREM2 can induce cytokine expression in THP-1 monocytes and macrophages, reinforcing the theory that it probably plays important roles in initiating inflammation and activating the cells. Interestingly, the concentration (1.0 μg/mL) of sTREM2 that was used to treat the cells in this study was over twenty times more concentrated than what is found in plasma and over a hundred times more than what is found in CSF but did not cause significantly aberrant inflammatory cytokine expression. This supports the notion that increased sTREM2 may not be recognized as pathogenic stimuli in THP-1 cells and macrophages but rather may have important regulating functions. Also, in support of this, was sTREM2′s ability to induce the expression of pro-inflammatory cytokines in THP-1 cells throughout the treatment time course but stimulate the expression of anti-inflammatory chemokines at later time points.

It has been shown that sTREM2 has a relatively short half-life of about 4 h in mouse brains [26], which supports the theory of 0.1 μg/mL of sTREM2 being capable of inducing expression of cytokines more so than 1.0 μg/mL. Although culture conditions and in vivo conditions differ largely, sTREM2’s half-life is probably longer in culture conditions. While the addition of sTREM2 was able to induce cytokine expression in cells, the underlying mechanisms or the signaling pathways involved remain to be defined.

### 4.2. The Effects of TREM2 Antibody on sTREM2-Induced Cytokine Expression

Recently, a TREM2 antibody known as AL002c, which is an agonist antibody, has been shown to activate microglia in 5XFAD mice, and administration of AL002c showed the induction of microglial proliferation in wildtype and R47H mutant TREM2 mice [27]. This shows that targeting TREM2 may be beneficial to modulating AD progression. We, therefore, tested whether a commercially available TREM2 antibody would be able to neutralize the ability of exogenous sTREM2 to induce cytokine expression in our cell model. Pre-incubation of sTREM2 with a rat anti-human TREM2 IgG_2B_ mAb was able to partially reduce the sTREM2-induced expression of inflammatory cytokines in THP-1 cells, but it significantly increased the expression of others. This antibody can bind to TREM2, but there is no evidence whether this antibody can activate TREM2 signaling in cells. Reduction in sTREM-induced expression of TNF-α and IL-10 at some time points mediated by the TREM2 antibody may suggest reduced inflammatory response as a result of neutralizing sTREM2-induced cytokine expression, but an increased expression in IL-1β suggests that other mechanisms may be at play. The complex of sTREM2/antibody may bind to certain receptors on the cell membrane, activating relevant signaling. TREM2 and sTREM2 possess a V-set domain, which allows binding to a large variety of substrates, and therefore, it is likely that sTREM2 signaling is strongly dependent on the substrates or receptors bound. Moreover, the effects of the antibody on sTREM2 are likely dependent on the epitope it binds. Interestingly, it was seen that the antibody alone was able to induce the expression of inflammatory cytokines, which was likely due to its engagement of the TREM2 receptor on the cell membrane, supporting its roles in the activation of cellular signaling. The fact that the NLRP-3 inhibitor can partially inhibit IL-1β expression induced by the complex of sTREM2+mAb suggests that the NLRP3 inflammasome pathway is activated and partially involved in the process of the sTREM2/mAb complex-mediated response.

### 4.3. The Effects of sTREM2 on Cytokine Expression in Polarized Macrophages

THP-1 cells were differentiated into macrophages to display the homeostatic phenotype (M0), pro-inflammatory phenotype (M1), or anti-inflammatory phenotype (M2). At early time points, the lower concentration of sTREM2 was able to stimulate the expression of pro-inflammatory cytokines in M0 and M2 macrophages but not M1 macrophages. TREM2 has been shown to play roles in macrophage activation in response to cerebral lesions and pathological stimuli [28]. While sTREM2′s effects on macrophage activation are largely uncharacterized, our results show that sTREM2 may promote a pro-inflammatory response in cells that are not “pro-inflammatory” at early stages of treatment. This suggests that sTREM2 likely plays important roles in activating cells and initiating inflammation. The literature has shown that TREM2 receptor activation attenuated neuro-inflammation in mouse AD models [29], and therefore, it would be expected that sTREM2 induces the expression of anti-inflammatory cytokines or modulates the microglial phenotype to help resolve primary inflammation for tissue repair. Our results show that sTREM2 induced an increased expression of TARC and IL-10 in M0, M1, and M2 cells at 8 h as compared to untreated macrophages at the later time points, except that TARC expression was reduced in M2 cells at 8 h. This suggests that while sTREM2 is capable of inducing the expression of inflammatory cytokines at an early stage, sTREM2 may also be able to upregulate the expression of anti-inflammatory cytokines at a later stage for the resolution of inflammatory responses in polarized macrophages, which is similar to the results obtained from THP-1 monocytes.

### 4.4. The Signaling Pathways Involved in sTREM2-Induced Cytokine Expression

We have shown that sTREM2 can modulate the expression of cytokines in THP-1 cells and in THP-1-derived macrophages. However, the signaling pathways that sTREM2 utilizes to induce cytokine expression have not been defined yet. TREM2 is known to signal through Syk, a tyrosine kinase, to mediate downstream signaling, which is recruited and phosphorylated by ITAMS with TREM2′s adaptor protein, DAP12 [30]. TREM2 has also been shown to antagonize toll-like receptor (TLR)-mediated inflammatory responses through the modulation of JNK and NFκB in microglia and macrophages [3,31]. Piceatannol is an inhibitor of Syk, which is the major signaling kinase for TREM2 receptor activation. However, this Syk inhibitor showed no effect on cytokine expression induced by sTREM2. This indicates that sTREM2 has distinct signaling mechanisms differing from the TREM2 receptor on THP-1 cells. NFκB plays important roles in the innate immune response and acts as a crucial regulator of IL-1β expression [32,33]. IL-1β has also been shown to increase neuronal susceptibility to degeneration [34]. MG-132 is a well-known inhibitor of NFκB, which inhibits proteasome-mediated IκB degradation and, therefore, NFκB activation. MG-132 was shown to partially reduce IL-1β expression, suggesting that NFκB is not the main signaling pathway responsible for sTREM2-induced cytokine expression. SP600125 was then used to inhibit JNK, which is downstream in the MAPK signaling pathway. Our results showed that SP6000125 inhibited the expression of the cytokines induced by sTREM2 in THP-1 cells to levels similar to controls. This indicates that in myeloid-derived cells, sTREM2 may primarily use the JNK–MAPK pathway to induce cytokine expression. It is likely that MAPK–JNK signaling is one of the pathways activated in the AD brain at an early stage since sTREM2 levels were shown to rise at this time [17]. Previously, the JNK–AP1 pathway was linked to the expression of inflammatory genes in the human AD brain and in human brain endothelial cells treated with Aβ peptide [20]. Moreover, there is supporting evidence that the JNK cascade is activated in AD neurons in response to Aβ [35]. A study also showed that intracellular Aβ accumulation activates JNK activation [36]. Together these results suggest that MAPK–JNK signaling may be one of the main pathways for sTREM2-induced cytokine expression in cells or behind chronic neuroinflammation in AD.

### 4.5. Shedding of sTREM2 by M0, M1, and M2 Macrophages

It is widely accepted that M2 macrophages express the highest levels of TREM2 followed by M0 macrophages, and M1 macrophages express the least amount of TREM2. It is currently unknown whether these phenotypes of macrophages actively shed sTREM2 into their surrounds. Here we showed that M2 macrophages shed the highest levels of sTREM2 into their surrounding environment followed by M0 macrophages and lastly by M1 macrophages. M0 and M2 macrophages showed an approximate doubling in the shedding sTREM2 from 24 to 48 h as compared to M1 macrophages. This confirms that the non-proinflammatory phenotypes express and shed the highest levels of sTREM2. sTREM2 may be an important molecule in initiating and regulating innate immune responses. To support this, if M0 and M2 macrophages are acting in a homeostatic or restorative manner, respectively, the continuous active shedding of sTREM2 into the surrounding environment may act as a decoy receptor to bind the ligands for the TREM2 receptor on cells or to serve as a molecule that recognizes, initiates, or terminates an appropriate immune response in macrophages or other cell types. Although sTREM2 was being shed in cell culture media to different degrees during our treatment, the percentage compared to our treatment with sTREM2 was minimal; therefore, we can rule out that it had any major effect on the experimental results.

## 5. Conclusions

In summary, sTREM2 at a lower concentration seems to sufficiently stimulate the expression of several pro-inflammatory cytokines throughout the time course, while sTREM2 at both a lower and a higher concentration appear to stimulate the expression of anti-inflammatory cytokines more consistently at later time points in THP-1 cells and macrophages. sTREM2 was also shown to differentially regulate cytokine expression in M0, M1, and M2 macrophages. This could possibly indicate that different concentrations and incubation times of sTREM2 may have differential effects on cytokine expression profiles in these cells. The co-incubation of sTREM2 with a TREM2 antibody neutralized the effects of sTREM2 on inducing the expression of some cytokines but promoting the expression of others (such as IL-1β). The increased expression of IL-1β induced by the sTREM2/TREM2 antibody complex is partially mediated through the NLRP3 inflammasome pathway. More importantly, the JNK inhibitor was capable of blocking sTREM2-induced cytokine expression, suggesting that sTREM2 regulates the expression of cytokines mainly via the MAPK–JNK pathway.

## Figures and Tables

**Table 1:** Human cytokine Multiplex ELISA results (pg/mL) for M0 macrophages, M1 macrophages, and M2 macrophages treated for 2 h by 0.1 μg/mL or 1.0 μg/mL sTREM2.

	IL-1α	IL-1β	IL-6	IL-8	GM-CSF	IFN-γ	MCAF	TNF-α
M0 CTR	25.58	28.01	9.47	183.83	111.45	28.14	31.08	101.89
M0 0.1 µg/mL sTREM2	22.97 ± 0.42	37.26 ± 2.79	12.7 ± 0.42	163.96 ± 30.11	92.11 ± 18.93	41.85 ± 22.71	34.63 ± 9.31	113.42 ± 8.78
M0 1 µg/mL sTREM2	29.64	36.09	10.93	165.38	99.53	41.45	31.58	104.44
M1 CTR	24.99	28.02	9.76	188.88	121.12	35.97	36.14	113.41
M1 0.1 µg/mL sTREM2	24.99	33.67	9.17	135.57	86.16	29.71	31.58	109.87
M1 1 µg/mL sTREM2	24.99	35.28	9.46	151.9	98.05	43.81	32.09	107.21
M2 CTR	24.99	36.9	10.64	110.74	87.64	43.8	125.28	117.84
M2 0.1 µg/mL sTREM2	26.16	38.52	9.76	109.32	77.97	53.98	125.29	124.04
M2 1 µg/mL sTREM2	34.28	41.75	10.94	128.48	119.63	79.83	133.38	120.5

## Data Availability

Data are available upon request.

## Supplementary Materials

The following supporting information can be downloaded at: https://www.mdpi.com/article/10.3390/biology13020087/s1, Figure S1: FACS analysis for binding of commercial anti-TREM2 (Human/mouse) antibody (monoclonal rat IgG_2B_, clone 237920; Cat#: MAB17291, R & D Systems) to THP-1 cells; Figure S2: SPR analysis for binding of commercial anti-TREM2 (Human/mouse) antibody (monoclonal rat IgG_2B_, clone 237920; Cat#: MAB17291, R & D Systems) to soluble human TREM2 (Sino Biological #11084-H08H); Figure S3: FACS analysis on markers for polarization of M0 into M1 macrophages; Figure S4: FACS analysis on markers for polarization of M0 into M2 macrophages; Figure S5: Standard curves for multiplex ELISA analysis of cytokine expression in M0, M1, and M2 macrophages; Figure S6: Standard curve for ELISA analysis of sTREM2 in culture media of M0, M1, and M2 macrophages at 24 h and 48 h; Table S1: Forward and reverse qPCR primers used for the detection of cytokine expression in this study.
