# Supplementary material for: sTREM2 Differentially Affects Cytokine Expression in Myeloid-Derived Cell Models via MAPK–JNK Signaling Pathway
Source: Biology (Basel). 2024 Jan 30;13(2):87. doi: 10.3390/biology13020087 (PMC10886855; doi:10.3390/biology13020087)
Supplement: Supplementary file 1 [file biology-13-00087-s001.zip › biology-2800201-supplementary.pdf]

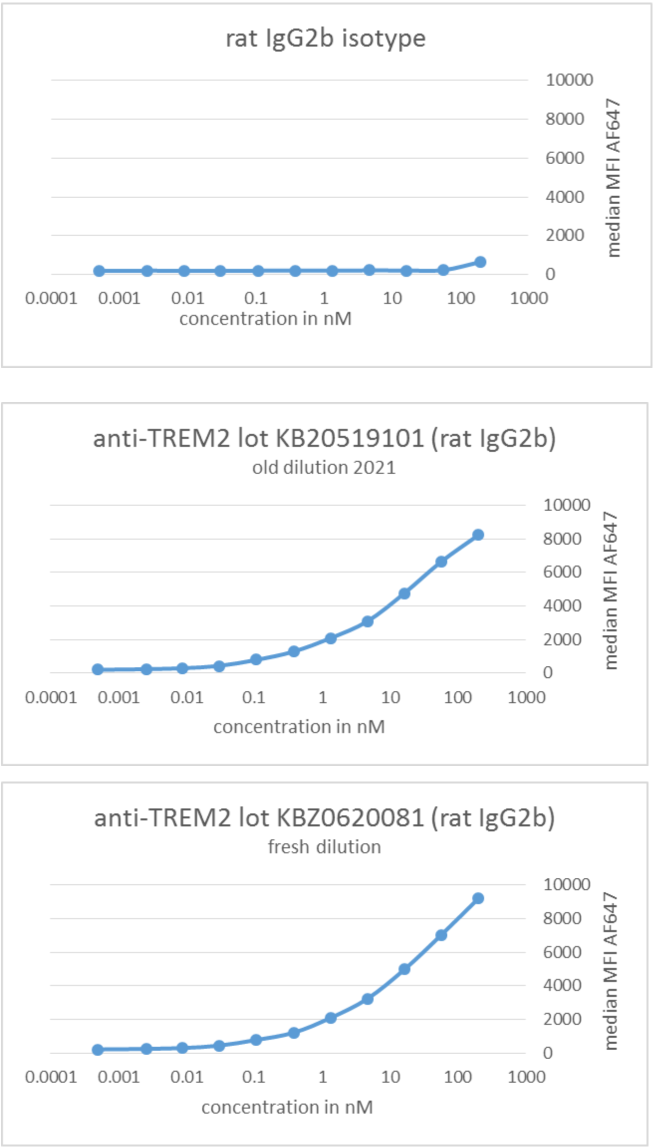

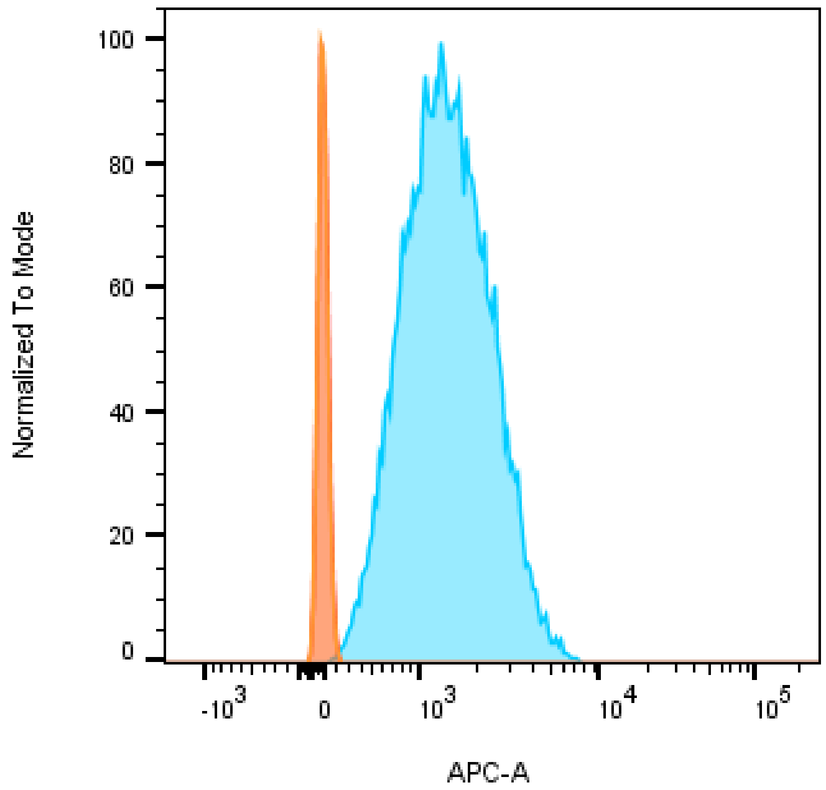

	Sample Name	Subset Name	Median : APC-A	Mean : APC-A
	unstained_A1_A01_001.fcs	Singlets	88,7	90,2
	Isotype_A2_A02_002.fcs	Singlets	89,9	92,9
	TREM2_A3_A03_003.fcs	Singlets	1376	1581

Supplementary Figure S1: FACS analysis for binding of commercial anti-TREM2 (Human/mouse) antibody (monoclonal rat IgG_{2B}, clone 237920; Cat#: MAB17291, R & D Systems) to THP-1 cells. A rat IgG2b isotype was used as a control for the assay.

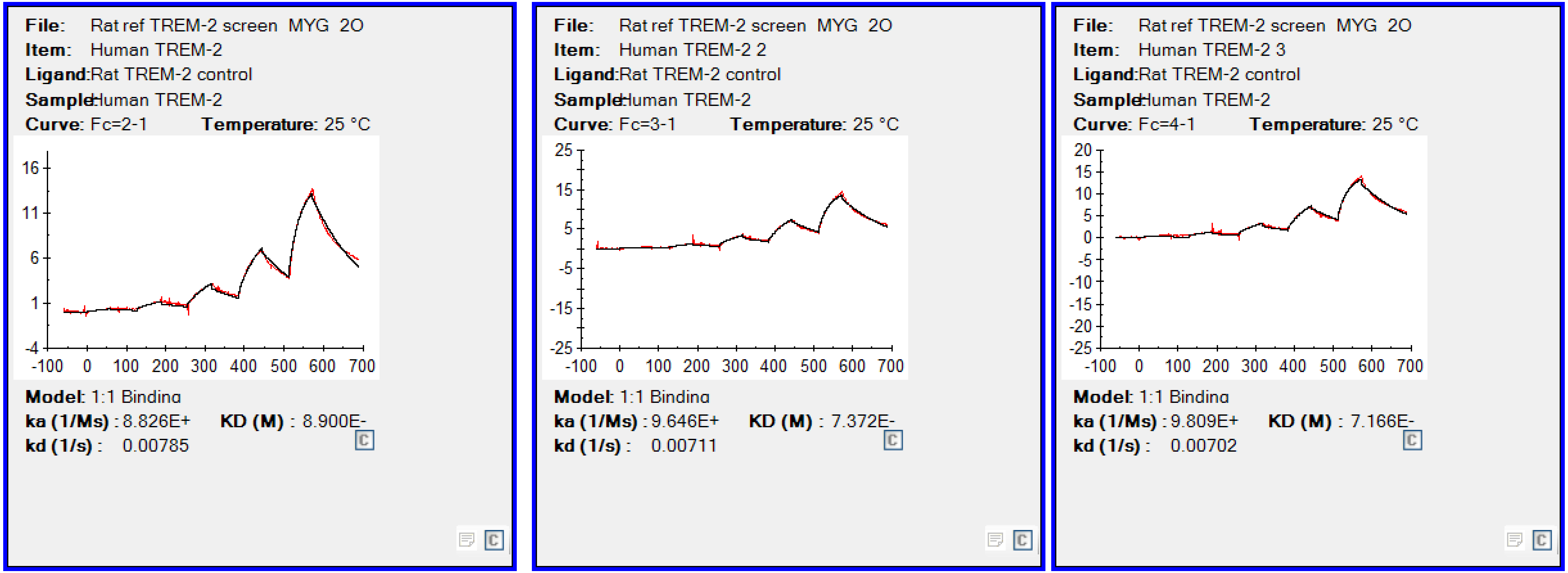

Supplementary Figure S2: SPR analysis for binding of commercial anti-TREM2 (Human/mouse) antibody (monoclonal rat IgG_{2B}, clone 237920; Cat#: MAB17291, R & D Systems) to soluble human TREM2 (Sino Biological #11084-H08H).

A). CD80

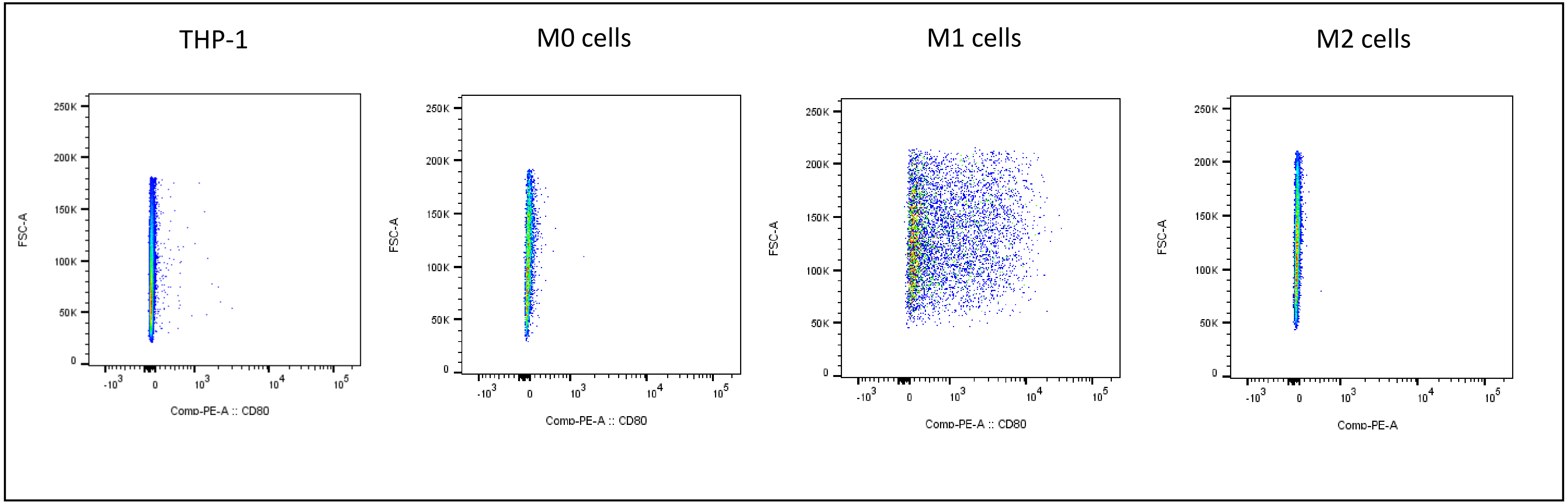

B). CD38

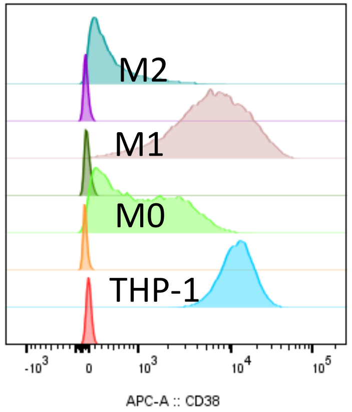

	Sample Name	Subset Name	Count	Median : APC-A
	M2_ CD38	Intact cells (FVS450 negative	13406	339
	M2 unstained	Intact cells (FVS450 negative	13802	53.9
	M1_ CD38	Intact cells (FVS450 negative	12889	5874
	M1 unstained	Intact cells (FVS450 negative	12640	71.9
	M0_ CD38	Intact M0 cells	14784	956
	M0 unstained	Intact M0 cells	14668	42.4
	THP-1_ CD38	Intact cells (FVS450 negative	7327	11429
	THP-1 unstained	Intact cells (FVS450 negative	7076	99.0

C). CD40

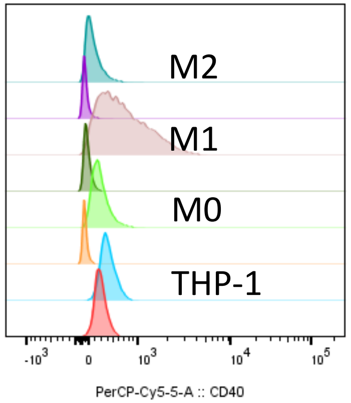

	Sample Name	Subset Name	Count	Median : PerCP-Cy5-5-A
	M2_ CD40	Intact cells (FVS450 negative)	14008	148
	M2_ isotype	Intact cells (FVS450 negative)	13875	44.9
	M1_ CD40	Intact cells (FVS450 negative)	12631	614
	M1_ isotype	Intact cells (FVS450 negative)	12888	70.6
	M0_ CD40	Intact M0 cells	14887	261
	M0_ isotype	Intact M0 cells	14761	41.1
	THP-1 CD40	Intact cells (FVS450 negative)	7400	399
	THP-1 isotype	Intact cells (FVS450 negative)	7291	282

Supplementary Figure S3: FACS analysis on markers for polarization of M0 into M1 macrophages. A). CD80 is a costimulatory molecule on immune cells and is recognized as a M1 cell marker. CD80 is expressed in M1 macrophages but not in THP-1, M0 and M2 cells. B). CD38 IS a marker of inflammatory macrophage and its expression is mostly detected in M1 macrophages. C). CD40 is a member of TNF receptor superfamily and is a marker of M1 macrophage. Its expression is detected in M1 macrophages but not in other types of cells.

A). CD206

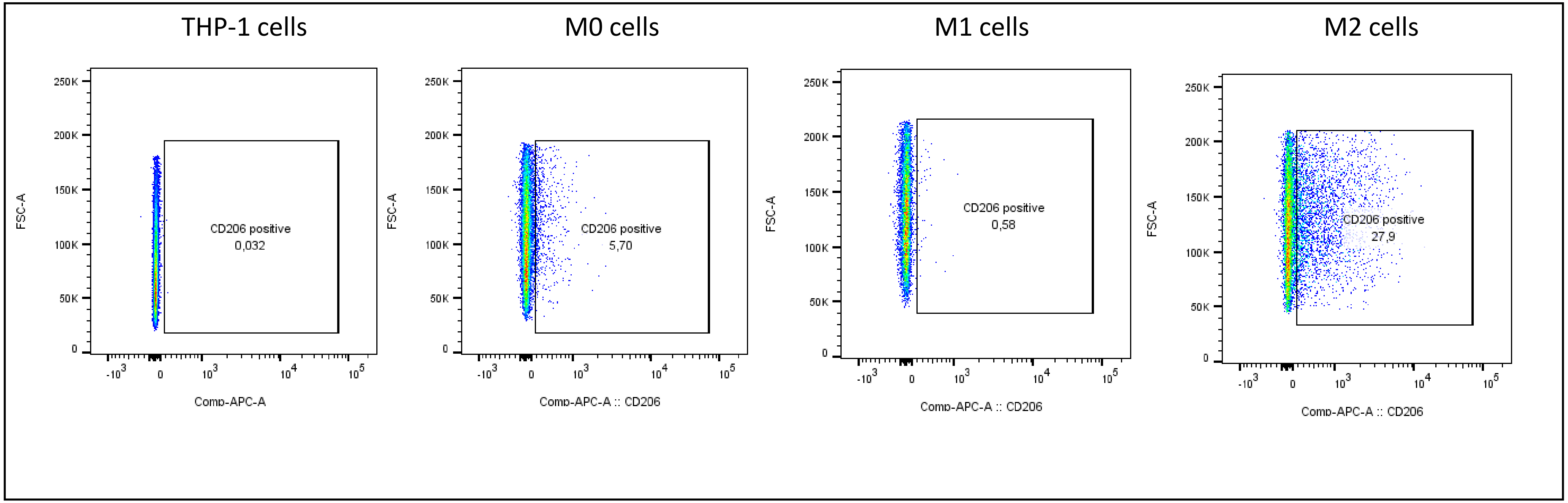

B). CD209

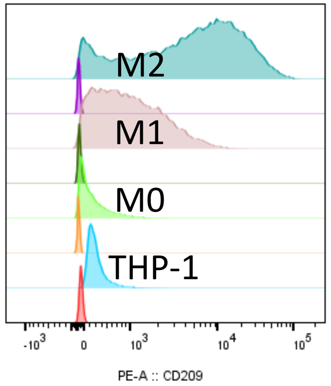

	Sample Name	Subset Name	Median : PE-A
	M2_ CD209	Intact cells (FVS450 negative)	5439
	M2 isotype	Intact cells (FVS450 negative)	20,5
	M1_ CD209	Intact cells (FVS450 negative)	893
	M1 isotype	Intact cells (FVS450 negative)	29,5
	M0_ CD209	Intact M0 cells	181
	M0 isotype	Intact M0 cells	16,7
	THP-1_ CD209	Intact cells (FVS450 negative)	274
	THP-1 isotype	Intact cells (FVS450 negative)	56,5

C). MS4A4A

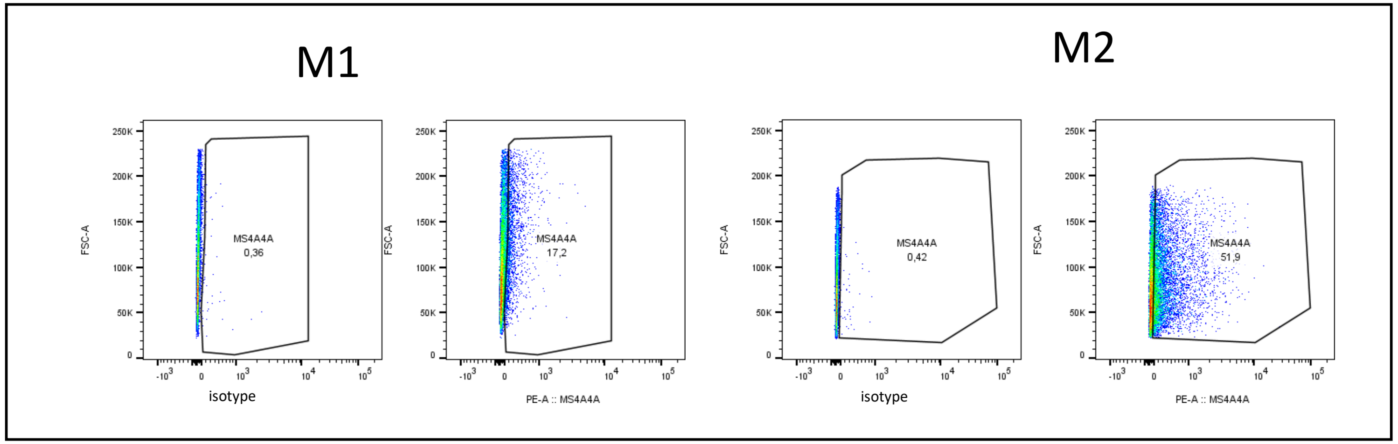

Supplementary Figure S4: FACS analysis on markers for polarization of M0 into M2 macrophages. A). CD206 is mannose receptor and is recognized as a M2 cell marker. CD206 is expressed in M2 macrophages. B). CD209 is an DC-SIGN/C-type lectin and its expression is mostly detected in M2 macrophages. C). MS4A4 is a tetraspan molecule expressed during macrophage differentiation and M2/M2-like polarization, and its expression is mostly detected in M2 macrophages.

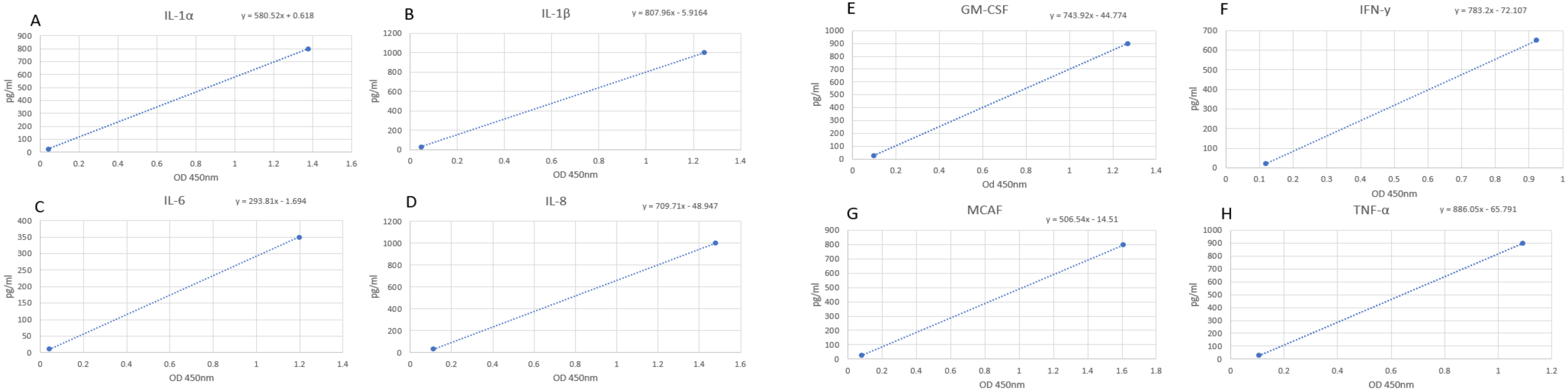

Supplementary Figure S5: Standard curves for multiplex ELISA analysis of cytokine expression in M0, M1, and M2 macrophages.

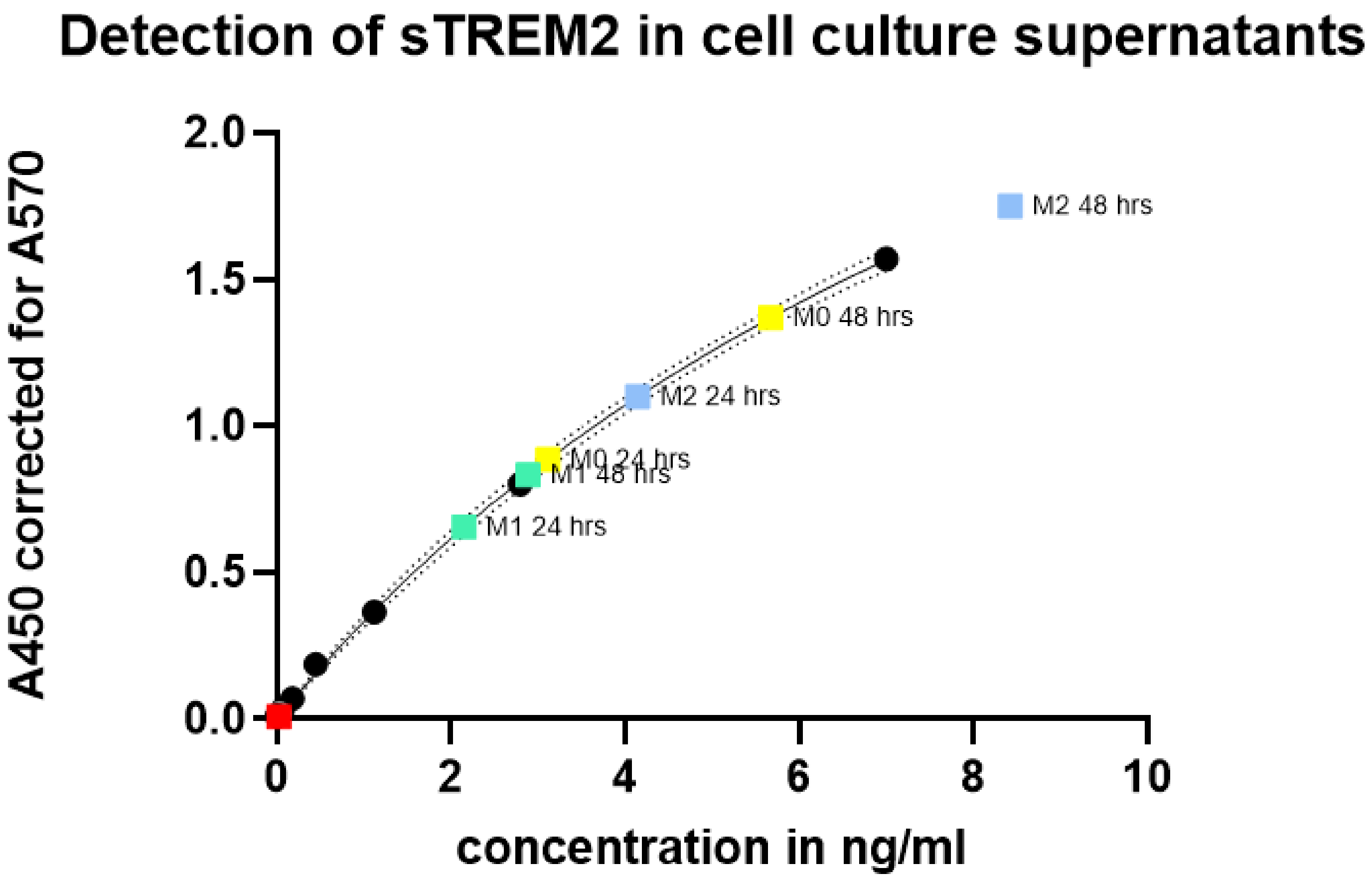

Supplementary Figure S6: Standard curve for ELISA analysis of sTREM2 in culture media of M0, M1, and M2 macrophages at 24hrs and 48 hrs.

Supplementary Table S1: Forward and reverse qPCR primers used for the detection of cytokine expression in this study.

Gene	5’ Primer (sense primer)	3’ Primer (anti-sense primer)
TNF-α	5’-GTGCTCCTCACCCACACC-3’	5’CCCTTCTCCAGCTGGAAGAC-3’
IL-1β	5’-CCTGTGGCCTTGGGCCTC-3’	5’-TGATGTACCAGTTGGGGAACT-3’
IL-10	5’-TATTTATTACCTCTGATACCTC-3’	5’-AATTATAATATTGGGCTTCTTTC-3’
IL-6	5’-AGTGAGGAACAAGCCAGAGC-3’	5’-AGCTGCGCAGAATGAGATGA-3’
TARC/CCL17	5’-AGTGAGGAACAAGCCAGAGC-3’	5’-AGCTGCGCAGAATGAGATGA-3’
β-actin	5’ – CATGTACGTTGCTATCGAGGC-3’	5’- CTCCTTAATACGCACGAT -3’
